# Hepatocyte nuclear factor-1beta enhances the stemness of hepatocellular carcinoma cells through activation of the Notch pathway

**DOI:** 10.1038/s41598-017-04116-7

**Published:** 2017-07-06

**Authors:** Jing-Ni Zhu, Lu Jiang, Jing-Hua Jiang, Xue Yang, Xiao-Yong Li, Jian-Xin Zeng, Rong-Yu Shi, Yang Shi, Xiao-Rong Pan, Zhi-Peng Han, Li-Xin Wei

**Affiliations:** 1 Tumor Immunology and Gene Therapy Center, Eastern Hepatobiliary Surgery Hospital, The Second Military Medical University, Shanghai, China; 2 Center of Digestive Endoscopy, Shandong University of Traditional Chinese Medicine Affiliated Hospital, Shandong, China; 3 Department of general surgery, Chinese PLA 82nd Hospital, Jiangsu, China; 4 Fujian Medical University, Fuzhou, China

## Abstract

Hepatocyte nuclear factor-1beta plays an important role in the development and progression of liver cancer. In recent years, the expression of HNF-1β has been reported to be associated with risk for a variety of cancers. The purpose of this study is to investigate whether the expression of HNF-1β promotes the malignancy of HCC and its mechanism. We retrospectively investigated the expression of HNF-1β in 90 patients with hepatocellular carcinoma and found that the high expression of HNF-1β indicated poor prognosis. We overexpressed HNF-1β in liver cancer cell lines and found the expression of liver progenitor cell markers and stemness were upregulated. The invasion ability and epithelial-mesenchymal transition (EMT)-associated genes were also significantly higher in liver cancer cells overexpressing HNF-1β than in the control group. A mechanistic study suggested the activation of the Notch signalling pathway probably plays a key role downstream of HNF-1β. More importantly, HNF-1β promoted tumourigenesis of HCC cells *in vivo*. In conclusion, high expression of HNF-1β not only promoted the de-differentiation of HCC cells into liver cancer stem cells through activating the Notch pathway but also enhanced the invasive potential of HCC cells and EMT occurrence, which would contribute to the enhancement of cell migration and invasion.

## Introduction

Primary liver cancer is one of the most common digestive system malignant tumours and causes high morbidity and mortality worldwide. Due to the lack of effective treatment, liver cancer prognosis is poor. According to the pathological type, primary liver cancers are divided into hepatocellular carcinoma (HCC), intrahepatic cholangiocarcinoma (ICC) and combined hepatocellular-cholangiocarcinoma (CHC) by the World Health Organization (WHO). The prognosis of the different pathological types of hepatocellular carcinoma is very different. The prognosis of ICC is significantly worse than that of HCC, due to tumour heterogeneity. If the difference between ICC and HCC is identified, then patients could be given more effective treatment^1, 2^. Therefore, it is necessary to explore the mechanisms of tumourigenesis in different pathological types of liver tumours.

Tumour heterogeneity is one of the characteristics of malignant tumours, which means different tumours or different tumour cells can show distinct morphological and phenotypic profiles, including cellular morphology, gene expression, metabolism, motility, proliferation, and metastatic potential. Many studies have indicated that the tumour heterogeneity is associated with cancer stem cells (CSCs)^3–6^. CSCs are a group of cells that exist in tumour tissue and have similar characteristics to stem cells, which have unlimited self-proliferation and differentiation potential to maintain tumour cell vitality^7^. Previously, studies have shown that liver CSCs may contribute to the high rate of recurrence and the heterogeneity of HCC^3, 7–9^. Several studies have shown that a portion of HCCs express the progenitor cell phenotype, such as CK7 and CK19^10^. Actually, this portion of liver cancer has a significantly worse prognosis and a higher recurrence rate after treatment^11^.

Our previous study found that tumour heterogeneity of primary hepatocellular carcinoma might be related to the expression of hepatocyte nuclear factor-1beta (HNF-1β)^12^. HNF-1β is a member of the hepatocyte nuclear factor family, which regulates the complex gene networks involved in lipid, carbohydrate, and protein metabolism^13^ and plays an important role in regulating liver development and hepatocyte differentiation. Previously, research on HNF-1β has mainly focused on a variety of diseases caused by HNF-1β mutations during organ development, such as maturity-onset diabetes of the young type 5 (MODY5), renal cysts, neonatal diabetes, pancreatic hypoplasia, abnormal liver function, cholestasis, etc.^14, 15^. HNF-1β down-regulation probably promotes fetal liver stem/progenitor cells differentiation into hepatocytes^16^. However, recent studies have shown that there is a clear correlation between HNF-1β and the occurrence and development of tumours. HNF-1β variants are associated with prostate and ovarian cancer activation^17–19^. The expression of HNF-1β was closely related to the prognosis of pancreatic cancer^20^. The expression of HNF-1β is significantly higher in ovarian clear cell carcinomas (OCCCs)^21^. In recent years, the importance of HNF-1β in liver cancer diagnosis and prognosis has been gradually recognized. HNF-1β was a novel prognostic marker independent of the Milan Criteria in transplantable hepatocellular carcinoma^22^. Our previous study showed that HNF-1β expression was associated with a pathological subtype of primary liver cancer. HCC with high HNF-1β expression exhibited the biliary phenotype and worse prognosis. However, the mechanisms for these differences remain unclear. In this study, we found that HCC cells with HNF-1β overexpression could present stronger stemness and hepatic stem cell properties. HNF-1β also increased the invasion potential and EMT of HCC cells.

## Results

### The expression of HNF-1β was negatively correlated with the prognosis of patients with HCC

**Figure 1:**
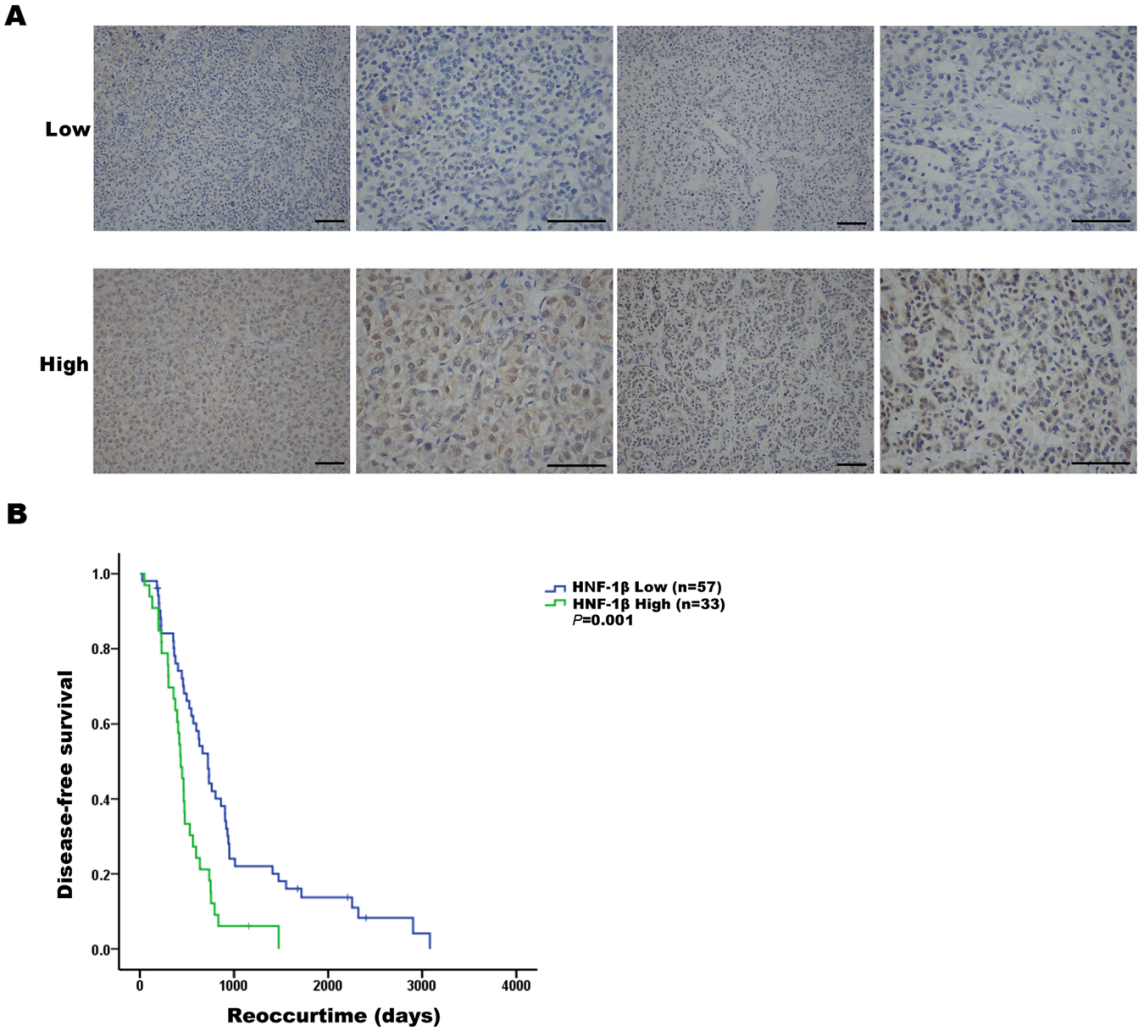
The expression of HNF-1β was negatively correlated with the prognosis of patients with HCC. (**A**) HNF-1β immunohistochemical staining patterns in HCC tissues. According to the HNF-1β expression level, HCC patients were divided into an HNF-1β low expression group (the top row) and high expression group (the bottom row). (scale bar = 100 μm). (**B**) Kaplan-Meier analysis of HNF-1β expression in HCC patients. Low expression of HNF-1β in HCC tissues was associated with prolonged disease-free survival. The P value calculated by the log-rank test is indicated (P = 0.001).

We retrospectively reviewed the liver tumour tissue specimens collected from 90 liver cancer patients and analysed the correlation between the prognosis and the HNF-1β expression level. Immunohistochemistry data showed that there were differences in HNF-1β expression in different HCC samples (Fig. 1A). In HCC patients, the expression of HNF-1β was significantly negatively correlated with disease-free survival (DFS). According to the Kaplan-Meier survival analysis, the HCC patients with high HNF-1β expression had a significantly poorer DFS. The median (95% CI) DFS of patients with low HNF-1β expression was 723.000 (596.246–849.754) days, while the median (95% CI) DFS of patients with high HNF-1β expression was 433.000 (366.600–499.400) days (P = 0.001) (Fig. 1B). In the HCC cohort, the following factors had a significant effect on DFS: higher tumour HNF-1β expression (P = 0.001), higher tumour size (P = 0.010), higher TNM stage (P = 0.005), higher Edmondson grade (P = 0.021), and cirrhosis (P = 0.001). In the univariate analysis, the factors with P < 0.05 were selected for further multivariate Cox proportional hazards analysis. Multivariate analysis showed that higher tumour HNF-1β expression (hazard ratio (HR): 2.406, 95% CI: 1.415, 4.092, P = 0.007), higher TNM stage (HR: 3.596, 95% CI: 1.388, 9.314), and higher Edmondson grade (HR: 1.776, 95% CI: 1.086, 2.907) were independent predictors of DFS (Table 1). These results indicated that the level of HNF-1β expression is negatively correlated with the disease-free survival of patients with HCC and that higher expression of HNF-1Β indicates a worse prognosis.

**Table 1:** Univariate and multivariate analysis of DFS in HCC patients.

Variable	Number	Univariate analysis	Multivariate	
		P value	HR (95% CI)	P value
Tumour HNF-1B expression				
Low vs High	57 vs 33	0.001^*^	2.406 (1.415,4.092)	0.007^*^
Tumour size				
<5 cm vs ≥ 5 cm	38 vs 52	0.010^*^		NS
Tumour number				
Single vs Multiple	92 vs 8	0.339		NA
Cirrhosis				
No vs Yes	24 vs 66	0.001^*^		NS
TNM stage				
I/II vs III	82 vs 8	0.005^*^	3.596 (1.388,9.314)	0.008*
HbsAg				
Negative vs Positive	74 vs 16	0.794		NA
Serum AFP				
<400 vs ≥400	67 vs 23	0.499		NA
Edmondson grade				
I/II vs III	62 vs 28	0.021^*^	1.776 (1.086,2.907)	0.022^*^
Encapsulation				
No vs Yes	26 vs 64	0.142		NA
Portal vein thrombosis				
No vs Yes	65 vs 25	0.147		NA
Microvascular invasion				
No vs Yes	35 vs 55	0.451		NA
Macrovascular invasion				
No vs Yes	85 vs 5	0.005^*^		NS

NA, not adopted; NS, not significant; HR, hazard ratio; Factors with p < 0.05 in univariate analysis were used for further multivariate analysis. *P < 0.05 was considered statistically significant.

### HCC cells with HNF-1β overexpression strongly expressed liver progenitor cell markers

**Figure 2:**
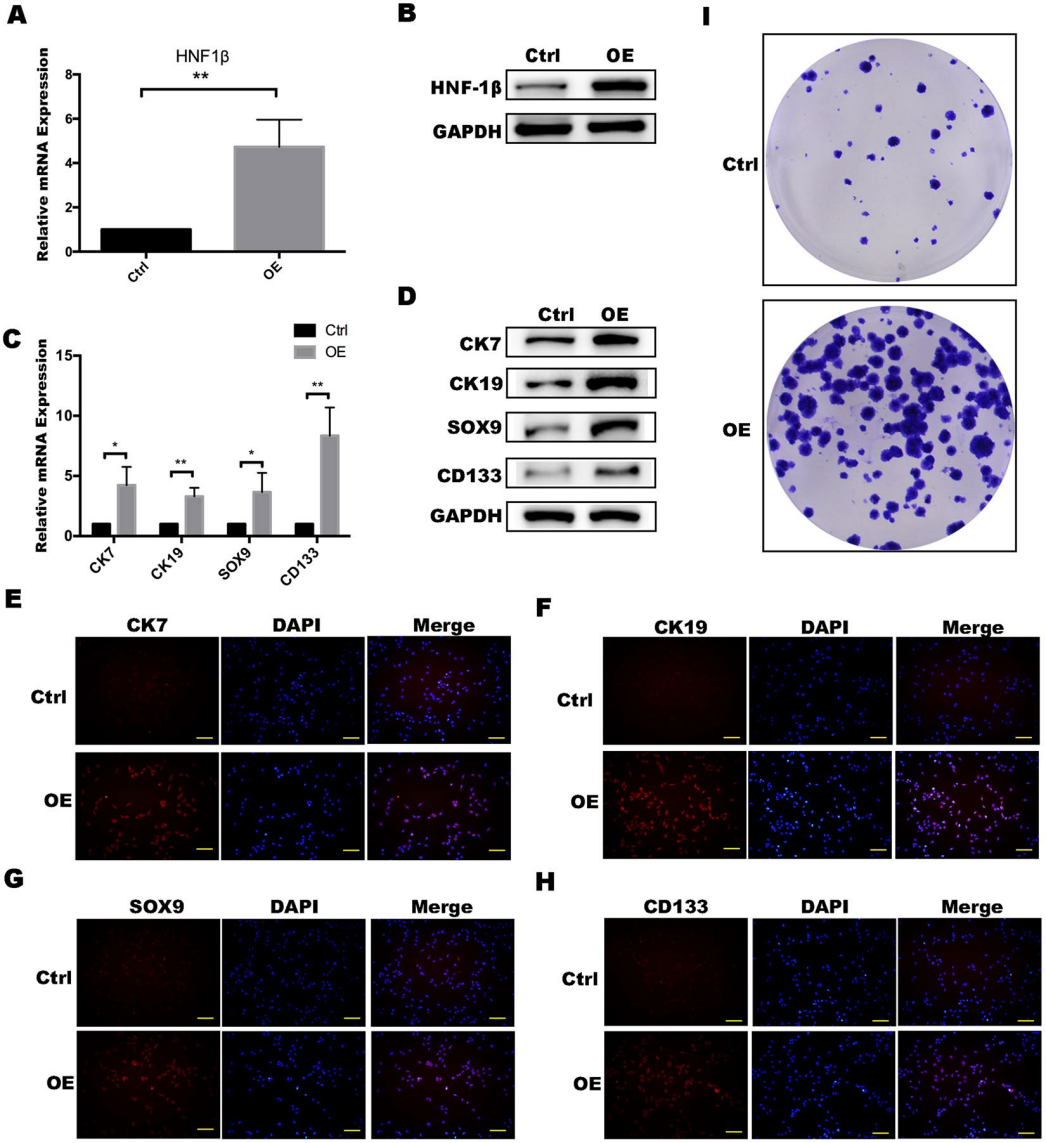
PLC/RF5 HCC cells with HNF-1β overexpression strongly expressed liver progenitor cell markers. (**A,B**) Detection of HNF-1β expression in HCC cells and HNF-1β-overexpressed HCC cells. OE: HNF-1β overexpression. (**C,D**) RT-PCR and western blotting were performed to detect the expression of the HPC markers (CK7, CK19, SOX9 and CD133). (*P < 0.05, **P < 0.01) Mean ± S.E.M. (**E–H**) Immunofluorescence staining was used to identify the expression level of the phenotypes (CK7, CK19, SOX9 and CD133) (scale bar = 100 μm). (**I**) A colony formation assay was used to detect the difference in the stemness between HCC cells and HNF-1β-overexpressed HCC cells.

In our previous study, we found that HNF-1β expression was positively correlated with the high expression of hepatic progenitor cell markers and the poor prognosis of HCC patients. To further demonstrate the role of HNF-1β in HCC, we employed HCC cell lines (PLC/RF5 and SMMU-7721 cells) as *in vitro* models. HNF-1β was stably transfected into the HCC cell lines to establish the HNF-1β stably overexpressing cell lines. The data from the SMMU-7721 cell line are shown in the supplemental data. Real-time PCR and western blotting showed that HNF-1β expression was obviously higher in the HNF-1β-overexpressing cell lines than in control (Fig. 2A,B and Supplemental Fig. 1A,B). As shown in Fig. 2C–H and Supplemental Fig. 1C–H, HNF-1β overexpression increased the expression of HPC markers in the HCC cell lines as expression of CK7, CK19, SOX9 and CD133 were dramatically increased. In the colony formation assay, there were significantly more clones in the HNF-1β overexpression group than in the control group (Fig. 2I and Supplemental Fig. 1I). These results demonstrated that an HNF-1β-positive malignant cell may retain some progenitor-like characteristics, and the correlation between the malignant degree of liver cancer and HNF-1β is probably due to upregulation of liver progenitor cell markers and the stemness of tumour cells.

### HNF-1β overexpression promoted invasion of HCC cells

**Figure 3:**
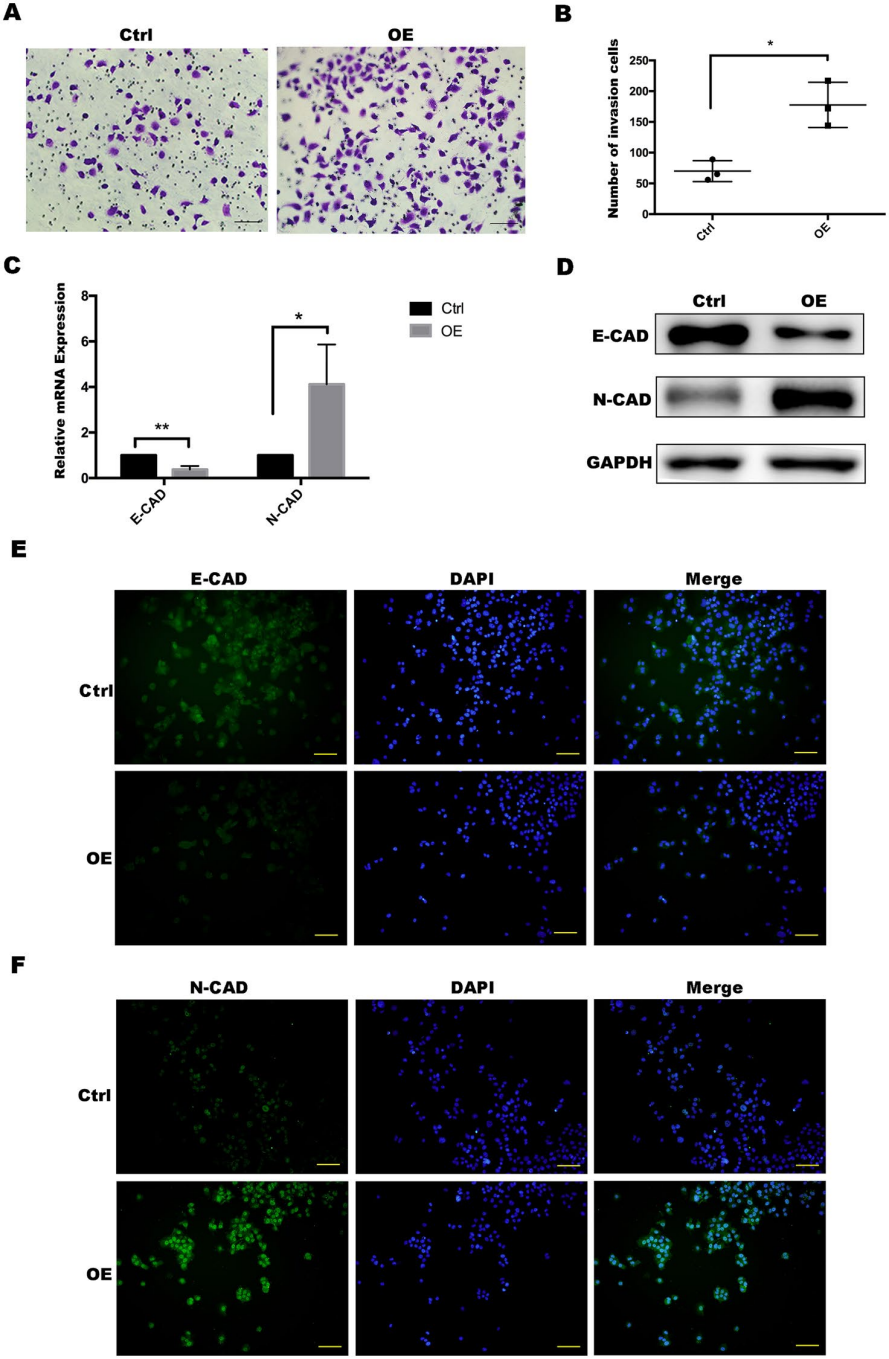
HNF-1β overexpression promoted invasion of PLC/RF5 HCC cells. (**A,B**) Transwell invasion assay was used to observe the invasion ability of the HCCs and the HNF-1β-overexpressed HCC cells. (**C,D**) RT-PCR and western blotting was performed to detect the expression of the EMT markers (E-cadherin and N-cadherin) in HCC cells and the HNF-1β-overexpressed HCC cells. (*P < 0.05, **P < 0.01) Mean ± S.E.M. (**E,F**) Immunofluorescence staining was used to identify the expression level of the EMT markers (E-CAD and N-CAD) (scale bar = 100 μm).

We then evaluated if HNF-1β overexpression would affect the invasive activity of HCCs. In the transwell invasion assay, the invasion ability of HCCs was higher in the HNF-1β overexpression group than in the control group (Fig. 3A,B and Supplemental Fig. 2A,B). The EMT plays an important role in increasing the invasiveness and migratory capacity of tumour cells. In real-time PCR and western blotting analysis, the expression of the cell adhesion protein E-CAD, an EMT marker, was reduced in HNF-1β overexpression HCC cells. Similarly, an increase in N-CAD expression was consistent with the above result (Fig. 3C,D and Supplemental Fig. 2C,D). There was a negative correlation between the E-CAD expression level and EMT ability, while the relationship between N-CAD and EMT ability was positively correlated. Immunofluorescence data showed the same trend as the above results (Fig. 3E,F and Supplemental Fig. 2E,F). These results indicated that HNF-1β played an important role in increasing the invasive activity of liver cancer cells.

### HNF-1β upregulated expression of Notch signalling-related genes

**Figure 4:**
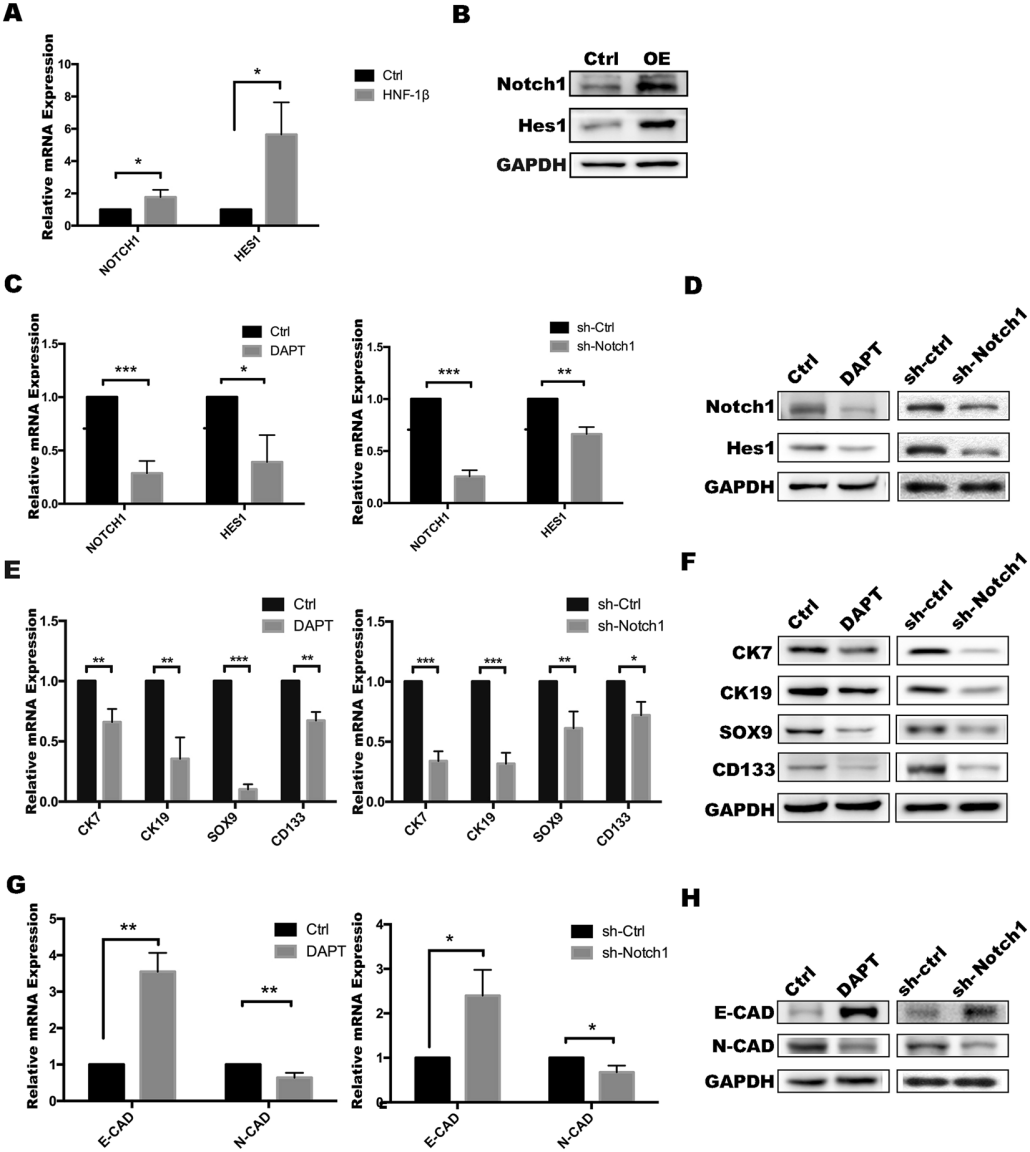
HNF-1β upregulated expression of Notch signalling-related genes in PLC/RF5 HCC cells. (**A,B**) The activation of NOTCH1 and HES1 in HCC cells was detected by RT-PCR and western blotting analysis. (**C,D**) The inhibition of the Notch pathway by DAPT or shRNA was confirmed using RT-PCR and western blotting. (**E,F**) RT-PCR and western blotting were used to determine the expression of CK7, CK19, SOX9 and CD133 after treatment with DAPT or Notch1 shRNA in HNF-1β-overexpressed HCC cells. (**G,H**) The expression of EMT markers was detected by RT-PCR and western blotting. (*P < 0.05, **P < 0.01, *** P < 0.001) Mean ± S.E.M. OE: HNF-1β overexpression. sh-Ctrl: negative control shRNA. sh-Notch1: Notch1 shRNA.

Recent studies have shown that the Notch signalling pathway plays an important role in the formation of the bile duct, which indicates that Notch signalling is probably involved in the conversion of HCC to cholangiocarcinoma^23, 24^. Meanwhile, the malignant degree of cholangiocarcinoma is higher than that of hepatocellular carcinoma, which indicates that the invasion and metastasis of cholangiocarcinoma is stronger and the stemness is higher than HCC. In recent years, accumulating evidence has indicated that EMT is closely linked to Notch activation. The Notch pathway is involved in the regulation of EMT^25^. Inhibition of the Jagged/Notch signalling pathway may inhibit EMT^26^. Targeting Notch1 could decreases HCC cell invasion *in vitro* ^27^. In addition, siRNA-mediated Notch1 silencing could suppress EMT^28^. Therefore, we conjectured that the Notch pathway is required for upregulation of stemness and EMT in response to HNF-1β overexpression and used western blotting to detect the activation of Notch1 and Hes1, the key molecules of the Notch pathway. Hes1 is the target downstream molecule in the Notch pathway and its increasing expression indicates that the pathway is activated. The results showed that compared with the expression of the Notch1 and Hes1 in control, HNF-1β overexpression significantly upregulated the Notch pathway markers in the HCC cell lines (Fig. 4A,B and Supplemental Fig. 3A,B). To prove the above result, the Notch pathway was inhibited in the HNF-1β-overexpressed HCC cell lines by treatment with N-[N-(3,5-difluorophenacetyl)–l–alanyl]–S-phenylglycine t-butyl ester (DAPT), a γ-secretase inhibitor, which inhibits the Notch pathway, or shRNA to silence Notch1, and then, the markers of stemness were measured and compared with those of the empty vector–transfected cells (Fig. 4C,D and Supplemental Fig. 3C,D). As shown in Fig. 4E,F and Supplemental Fig. E,F, the expression of HPC markers in the DAPT group and shRNA group was significantly lower than that in the HNF-1β overexpressed HCC cells, which suggested that Notch signalling is important downstream of HNF-1β for the expression of biliary/HPC markers. Meanwhile, Notch pathway suppression was accompanied by downregulation of EMT capacity (Fig. 4G,H and Supplemental Fig. 3G,H). These results strongly supported that HNF-1β could enhance the stemness of hepatocellular carcinoma cells through the Notch pathway.

### HNF-1β overexpression enhanced the ability of HCC cells to form a tumour ***in vivo***

**Figure 5:**
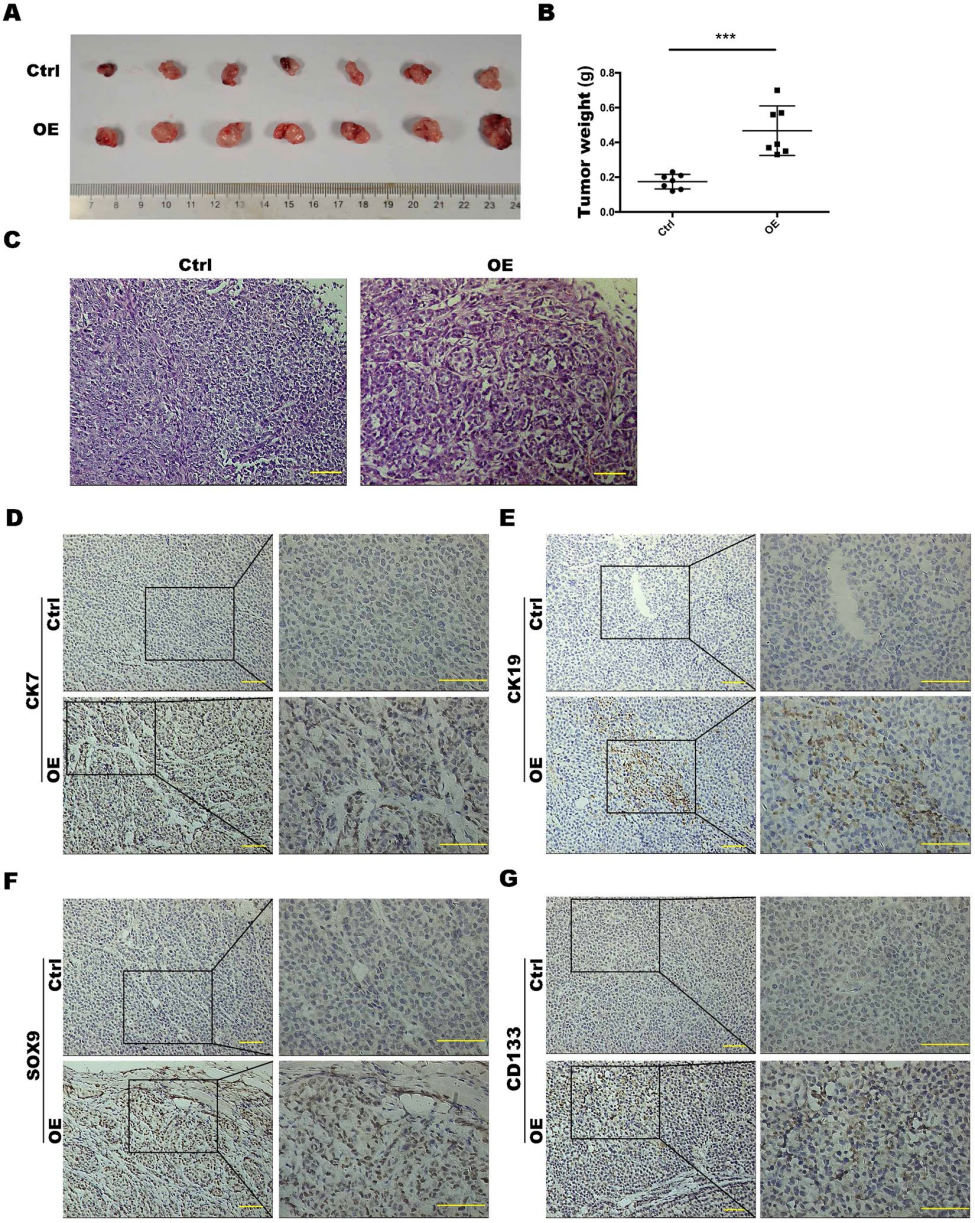
HNF-1β overexpression enhanced the ability of HCC cells to form tumours *in vivo*. (**A,B**) Tumour size of nude mice injected with PLC/RF5 HCC cells or HNF-1β-overexpressed PLC/RF5 cells (P < 0.001, mean ± S.E.M). (**C**) Representative H&E images of tumours. (**D–G**) Representative IHC images of CK7, CK19, SOX9 and CD133 of tumours in the Ctrl group and HNF-1β-overexpressed group (scale bar = 100 μm). n = 7.

In the above study, we found that HNF-1β increased the stemness and ability of invasion in HCC cells through upregulating the Notch pathway. To further elucidate the effect of HNF-1β on tumour formation *in vivo*, normal HCC cells or HNF-1β overexpression HCC cells were subcutaneously transplanted into nude mice; each group included seven nude mice. After four weeks, tumours were detected in all nude mice. The tumour size of the nude mice injected with HNF-1β overexpression HCC cells was significantly larger than that of the control group (Fig. 5A,B). In addition, IHC showed that the expression of HPC markers (CK7, CK19, SOX9 and CD133) was higher in the tumour formed by HNF-1β overexpression HCC cells than the control group (Fig. 5C–G). Overall, the above observations confirmed that HNF-1β enhanced the tumourigenic ability of HCC cells by regulating the stemness of HCC cells.

## Discussion

HCC has long been accepted to arise from hepatocytes, while ICC is derived from the intrahepatic biliary epithelium^29^. The fate relationship among HCC, ICC, and CHC is a matter of debate. Whereas lineage tracing data support the idea of ductal progenitor-like cells giving rise to hepatocytes^30^, there is also evidence that hepatocytes might give rise to cells of the ductal lineage. As such, periportal hepatocytes in patients with cholestatic or biliary autoimmune disorders can express biliary-specific markers^31^, whereas data from a mouse model of hepatocyte fate tracing support the observation that ICC can originate from fully differentiated hepatocytes. There is also evidence that HCC with biliary/HPC markers has a poor outcome. Additionally, hepatocyte transplantation in the rat supports the possibility of hepatocyte “trans-differentiation” into ductal cells^32^. More recently, some findings have shown that dual NOTCH and AKT signalling in hepatocytes can lead to their conversion into biliary cells that eventually progress into cholangiocarcinomas, a malignancy typically associated with a ductal origin^23, 33^.

In our previous study, we found that cholangiocarcinoma-like HCC (CLHCC), a novel HCC subtype that highly expresses HNF-1β and expresses cholangiocarcinoma-like traits and stem cell-like expression traits, tended to show poorer surgical outcome. HNF-1β is involved in biliary differentiation and is known as a biliary marker^12^. Our previous study also confirmed this view. In this study, we found that HNF-1β expression was associated with the pathologic subtype of primary liver cancer, with high expression in ICC and significantly lower expression in HCC. The malignant degree of ICC was recognized to be higher than that of HCC, and the prognosis was worse. Therefore, we considered whether HNF-1β influenced the prognosis of patients with liver cancer. In this study, our data suggests that HCC cells with HNF-1β overexpression have the potential to give rise to cells that molecularly and functionally resemble liver progenitors or “oval” cells. We found that liver cancer patients with increased HNF-1β expression had elevated stemness. Furthermore, the expression of HNF-1β was positively correlated with the prognosis of the liver cancer patients. Based on the above conclusions, we overexpressed HNF-1β in human liver cancer cell lines and detected the expression of HPC and EMT markers. We found that the invasion and malignance of the overexpressed group were significantly higher than the control. More recently, some findings have shown that dual Notch and AKT signalling in hepatocytes can lead to their conversion into biliary cells that eventually progress into cholangiocarcinomas, a malignancy typically associated with a ductal origin^23, 33^. In addition, many articles have reported that the Notch pathway and downstream Hes1 may regulate the differentiation of cholangiocarcinoma^33–38^. Certain gene deletions or mutations can result in a nuclear translocation of N2ICD, which triggers the Notch pathway, maintaining the stemness of the liver CSCs. In recent years, accumulating evidence has indicated that EMT is closely linked to Notch activation^26, 39^. Giovannimi C *et al*. discovered that both Notch1 and E-Cadherin contribute to invasion in HCC, indicating that the Notch1-E-Cadherin pathway in the tumour might mediate HCC recurrence and invasion^27^. Finally, the Notch pathway activation level was consistent with the clinical severity and prognosis of HCC patients^40^. Therefore, we investigated the Notch pathway and found that HNF-1β had the ability to activate Notch signalling in HCCs, which is also thought to be closely related to the occurrence and development of ICC. Overall, HNF-1β maintains the stemness of liver cancer cells by regulating the Notch signalling pathway and EMT, thereby enhancing the invasion and metastasis of liver cancer and worsening the prognosis of liver cancer patients. These studies raise the possibility that hepatocytes are inherently plastic and might participate in liver repair not only by self-duplication but also by dedifferentiation into progenitor cells.

Overall, research on the effect of HNF-1β on the maintenance of stemness in HCC cells and its mechanism could provide a useful target for prognostic prediction in HCC and provides a potential target for effective treatment of patients with primary liver cancer. However, whether HCC transdifferentiates to form ICC remains unclear as there was no morphological or pathological evidence observed in our experiments to support or refute this hypothesis. If this occurs, the mechanism remains unclear and requires further investigation.

## Materials and Methods

### Tissue samples

Liver cancer tissue samples were obtained from patients who had received a hepatectomy at the Department of Surgery, Eastern Hepatobiliary Surgery Hospital of the Second Military Medical University from 1997 to 2007. After surgery, the patients were followed up once every three months. Prior informed consent was obtained, and the study protocol was approved by the Ethics Committee of the Second Military Medical University. All experimental protocols were conducted in accordance with the Declaration of Helsinki of the World Medical Association.

### Animal model

Male BALB/C mice (6 weeks old, weighting 20~23 g) were obtained from Shanghai Laboratory Animal Center and housed in pathogen-free conditions. Animal protocols were approved by the Second Military Medical University Animal Care Committee. To investigate the effect of HNF-1β on tumour formation, cells were subcutaneously implanted in nude mice. Seven mice were used in each group.

### Cells and treatments

We used two kinds of human liver tumour cell lines, PLC/RF5 and SMMU-7721. The human liver tumour cell lines PLC/RF5 and SMMU-7721 (2 × 10^5^) were maintained in DMEM (GIBCO, Invitrogen, Carlsbad, CA, USA) containing 10% foetal bovine serum (FBS; GIBCO, Invitrogen), 100 units/mL penicillin and 100 mg/ml streptomycin. The cells were planted on six-well plates and incubated in a humidified incubator under 95% air and 5% CO_2_ at 37 °C. After 24 h, the cell culture medium was added with lentiviruses overexpressing HNF-1β. The medium was replaced after 24 h. The efficiency of transfection was highest after 72 h in the incubator.

### Generation of overexpressing cell lines

Lentivirus overexpressing HNF-1β or containing a control vector was purchased from Obio Technology (Shanghai, China). PLC/RF5 and SMMU-7721 cells were cultured in 6-well plates. After reaching 70% confluence, medium containing lentiviruses and polybrene (8 µg/ml; Hanbio) was added at a multiplicity of infection (MOI) of 10 and mixed with the cells. Polybrene was used to improve infection efficiency. After incubation for 24 h, the supernatants in the wells were replaced by DMEM containing FBS and cultured for 24 and 48 h for subsequent analyses.

### Immunofluorescence (IF)

Cells were cultured on glass coverslips, fixed with 4% paraformaldehyde, then permeabilized in TBS containing 0.4% Triton X-100, and blocked with 1% bovine serum albumin (BSA). Cells were then incubated with the primary antibodies overnight at 4 °C. The glass coverslips were washed with PBS-Tween and incubated with a fluorescent antibody as the secondary antibody for 1 h at 37 °C. Then, the glass coverslips were washed with TBS and stained with DAPI.

### Evaluation of the immunohistochemical staining

Two independent pathologists who were not informed of the patient’s characteristics assessed the immunohistochemical staining. All of the slides were observed and photographed with an Olympus microscope (IX-70 OLYMPUS, Japan). We used the following criteria to classify and analyse the clinical data: 0 for <5%, 1+ for 5% to 25%, 2+ for 25% to 50%, and 3+ for ≥50%. Cases were considered positive for HNF-1B when nuclear staining was observed in at least 5% (>1+) of the examined tumour cells. Based on the HNF-1β expression, the 90 HCC patients were divided into a high HNF-1β group (2+, 3+) and low HNF-1β group (1+, 0).

### Colony formation assay

Cells were planted in a six-well plate (5 × 10^2^ cells/well). After incubation for 14 days, the cells were washed with PBS, fixed with 4% paraformaldehyde, and stained with 0.1% crystal violet solution. The number of cell colonies containing more than 20 cells was counted under a microscope.

### Transwell invasion assay

To conduct the transwell assay, Matrigel invasion chambers installed with an 8.0-μm PET membrane in 24-well plates (Corning, USA) were used. The Matrigel invasion chambers was pre-treated with 50 μl DMEM without serum and incubated at 37 °C for 30 minutes. Cells (2 × 10^5^) in 15 μl DMEM without serum were plated in each upper chamber, while 500 μl DMEM containing 10% FBS was added in the lower chamber as a chemoattractant. After 36 h of conventional incubation, the upper surface cells were wiped with a cotton swab. The cells that invaded through the pores to the lower surface of the filter were fixed in 4% formaldehyde for 10 minutes and then stained with 0.1% crystal violet dye for 30 minutes. Stained cells were observed under a microscope.

### *In vivo* tumourigenicity experiments

Six-week-old male nude mice were obtained from Shanghai Experimental Animal Center, Chinese Academy of Science. Mice were maintained under a pathogen-free condition and treated in accordance with the institutional animal welfare guidelines of the Second Military Medical University. To assay the tumourigenicity, PLC/RF5 cells were collected, washed, and then suspended in PBS at a concentration of 1 × 10^6^ live cells/ml. The cell mixture (0.1 ml/mouse) was injected subcutaneously into the left armpit of mice. At the end of 4 weeks, the mice were sacrificed. The tumours were weighed.

### Real-time PCR

Total RNA was isolated using TRIZOL (Invitrogen, Carlsbad, CA, USA) according to the manufacturer’s protocol. RNA was quantified using an ND-2000 spectrophotometer (Nanodrop Technologies, Wilmington, DE), and complementary DNA synthesis was performed using the PrimeScript RT reagent Kit (Takara, Kyoto, Japan). Standard RT-PCR was conducted using a SYBR Green PCR Kit (Applied BI) according to the manufacturer’s instructions. The sequences of the PCR primers are shown in Table 2.

**Table 2:** The sequences of the PCR primers.

The primers used in this experiment.		
GAPDH	Forward	GGAGCGAGATCCCTCCAAAAT
	Reverse	GGCTGTTGTCATACTTCTCATGG
HNF1B	Forward	CACCAACATGTCTTCAAGTAAACAG
	Reverse	TTGTTGCGCACGAAGTAAGT
CK7	Forward	GGAAGCTATTCTGACATCACTTTC
	Reverse	CAGCCACCACCCACAATC
CK19	Forward	TGAGTGACATGCGAAGCCAAT
	Reverse	CTCCCGGTTCAATTCTTCAGTC
SOX9	Forward	ACGGCTCCAGCAAGAACAAG
	Reverse	CCCGTTCTTCACCGACTTCC
CD133	Forward	TTCTTGACCGACTGAGACCCA
	Reverse	TCATGTTCTCCAACGCCTCTT
E-cadherin	Forward	CGAGAGCTACACGTTCACGG
	Reverse	GGGTGTCGAGGGAAAAATAGG
N-cadherin	Forward	AGCCAACCTTAACTGAGGAGT
	Reverse	GGCAAGTTGATTGGAGGGATG
Notch1	Forward	GAGGCGTGGCAGACTATGC
	Reverse	CTTGTACTCCGTCAGCGTGA
Hes1	Forward	TCAACACGACACCGGATAAAC
	Reverse	GCCGCGAGCTATCTTTCTTCA

### Western blotting analysis

Cells were washed with PBS and lysed in cell lysis buffer for western blotting with 1 mM PMSF. Equal amounts of proteins were separated by SDS-PAGE and transferred to a nitrocellulose membrane. After transfer, the membrane was blocked in 5% fat-free milk/1 × TBS/0.1% Tween-20 for 1 h at room temperature and incubated with primary antibodies with gentle agitation overnight at 4 °C. The membrane was then incubated with the goat anti-mouse or antirabbit IgG secondary antibody (1:10,000) for 1 h at room temperature, followed by extensive washing with 1 × TBS/0.1% Tween-20. Immunoblots were developed by using the BeyoECL (Beyotime) and Tanon 5200 system.

### Statistical analysis

Statistical analysis was performed using SPSS software version 12.0 (SPSS, Chicago, IL). The DFS was calculated using the Kaplan-Meier method, and differences in survival rate were compared using a log-rank test. Univariate and multivariable analyses were based on the Cox proportional hazards regression model. P < 0.05 was considered significant.

## Electronic supplementary material

Supplementary Information
