# Supplementary material for: Hepatocyte nuclear factor-1beta enhances the stemness of hepatocellular carcinoma cells through activation of the Notch pathway
Source: Sci Rep. 2017 Jul 6;7:4793. doi: 10.1038/s41598-017-04116-7 (PMC5500528; doi:10.1038/s41598-017-04116-7)
Supplement: Supplementary file 1 — Supplementary Information [file 41598_2017_4116_MOESM1_ESM.doc]

**Hepatocyte nuclear factor-1beta enhances the stemness of hepatocellular carcinoma cells through activation of the Notch pathway**

**Authors:** Jing-Ni Zhu^a,1^, Lu Jiang^b,1^, Jing-Hua Jiang^a,1^, Xue Yang^a^, Xiao-Yong Li^a^, Jian-Xin Zeng^d^, Rong-Yu Shi^d^, Yang Shi^c^, Xiao-Rong Pan^d^, Zhi-Peng Han^a,*^＆Li-Xin Wei^a,*^


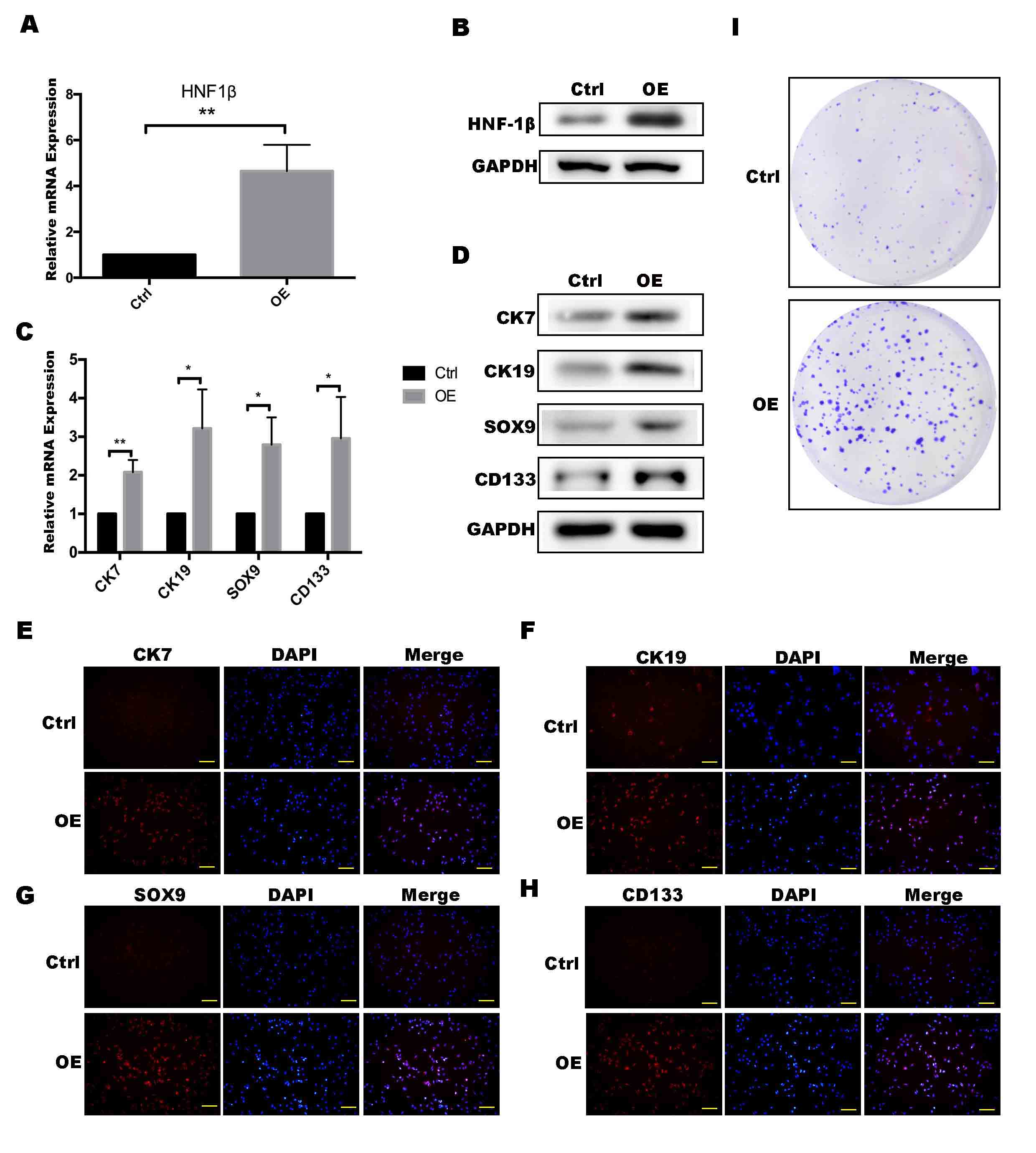


**Supplemental Figure 1. SMMU-7721 HCC cells with HNF-1β overexpression strongly expressed liver progenitor cell markers.**

(A-B) Detection of HNF-1β expression in HCC cells and HNF-1β-overexpressed HCC cells. OE：HNF-1β overexpression. ­­

(C-D) RT-PCR and western blotting were performed to detect the expression of the HPC markers (CK7, CK19, SOX9 and CD133). (*P < 0.05, **P < 0.01) Mean ± S.E.M.

(E-H) Immunoﬂuorescence staining was used to identify the expression level of the phenotypes (CK7, CK19, SOX9 and CD133) (scale bar = 100 μm).

(I) Colony formation assay was used to detect the difference in the stemness between HCC cells and HNF-1β-overexpressed HCC cells.


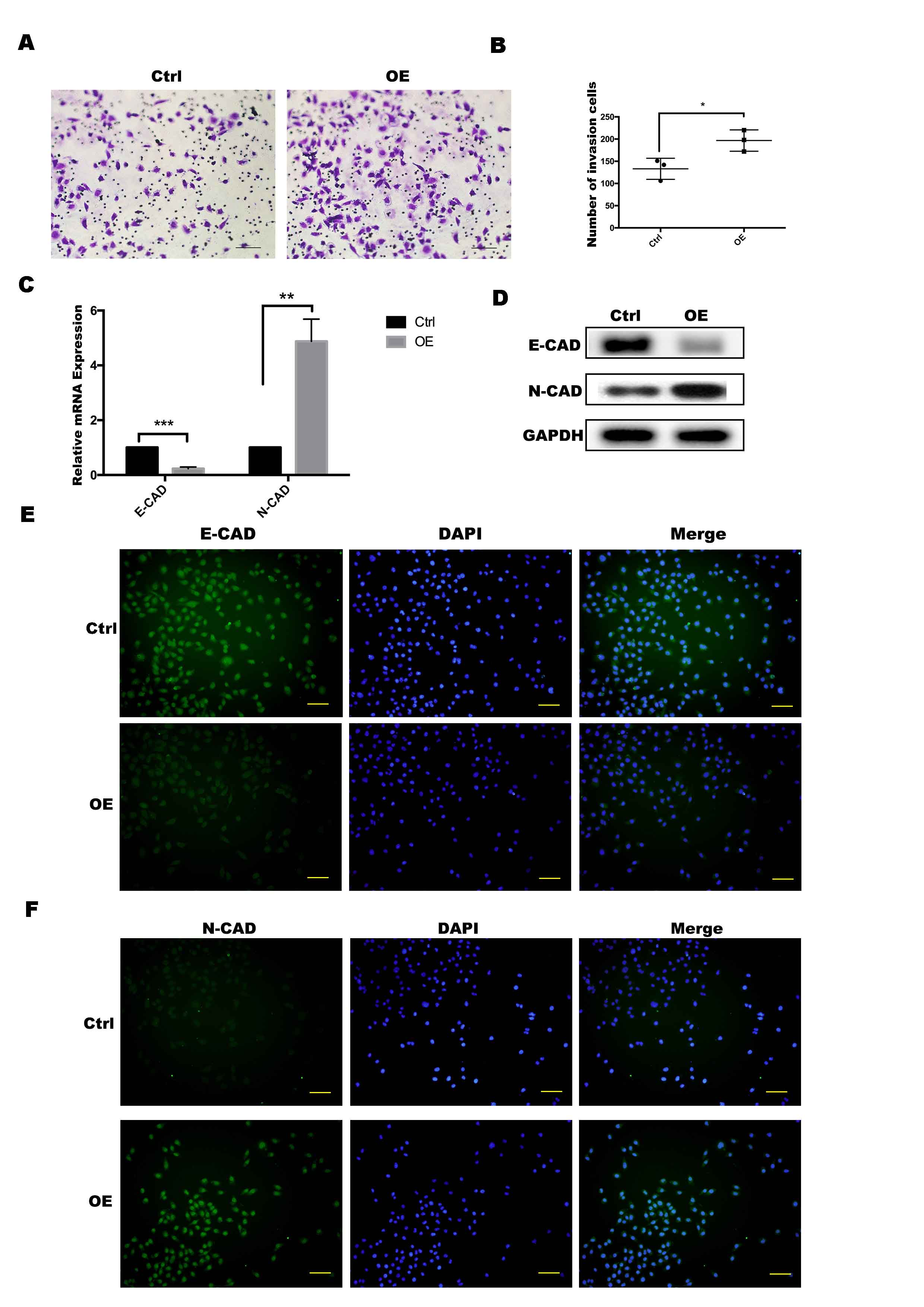


**Supplemental Figure 2. HNF-1β overexpression promoted invasion of SMMU-7721 HCC cells.**

(A-B) A transwell invasion assay was used to observe the invasion ability of the HCCs and the HNF-1β-overexpressed HCC cells.

(C-D) RT-PCR and western blotting were performed to detect the expression of the EMT markers (E-cadherin and N-cadherin) in HCC cells and the HNF-1β-overexpressed HCC cells. (*P < 0.05, **P < 0.01) Mean ± S.E.M.

(E-F) Immunoﬂuorescence staining was used to identify the expression level of the EMT markers (E-CAD and N-CAD) (scale bar = 100 μm).


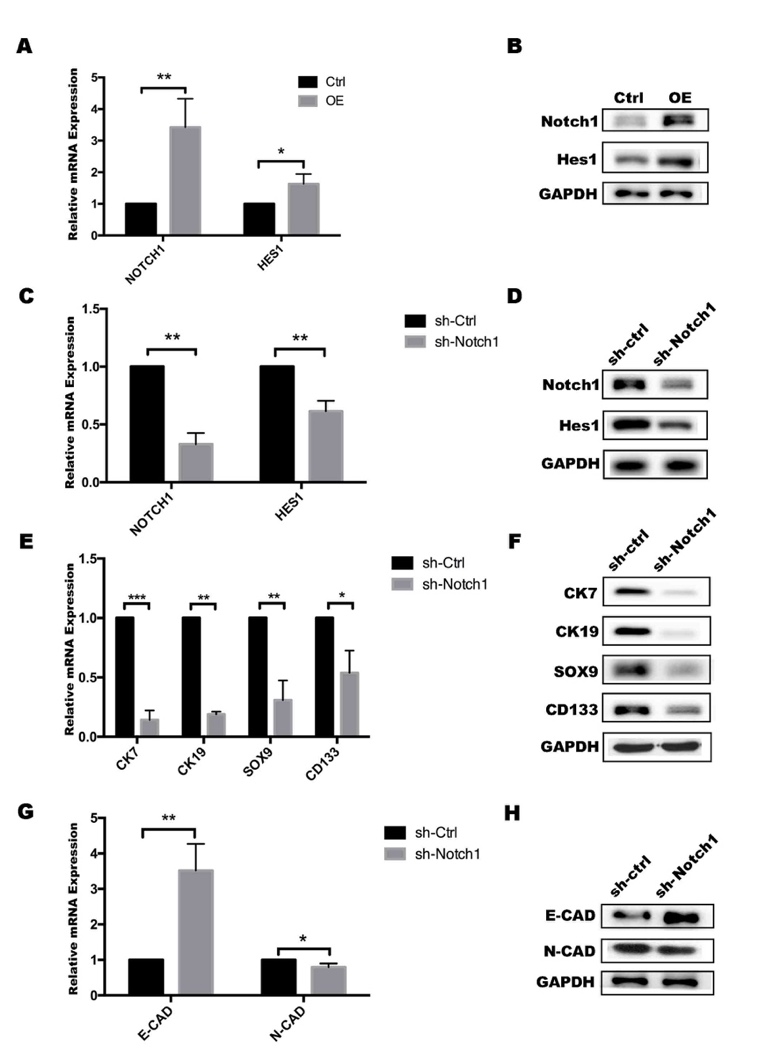


**Supplemental Figure 3. HNF-1β upregulated expression of Notch signalling-related genes in SMMU-7721 HCC cells.**

(A-B) The activation of NOTCH1 and HES1 in HCC cells was detected by RT-PCR and western blotting analysis.

(C-D) The inhibition of the Notch pathway by shRNA was conﬁrmed using RT-PCR and western blotting.

(E-F) RT-PCR and western blotting were applied to determine the expression of CK7, CK19, SOX9 and CD133 after treatment with Notch1 shRNA in HNF-1β-overexpressed HCC cells.

(G-H) The expression of EMT markers was detected by RT-PCR and western blotting. (*P < 0.05, **P < 0.01, *** P < 0.001) Mean ± S.E.M. sh-Ctrl: negative control shRNA. sh-Notch1: Notch1 shRNA.
